# Prescribing Patterns of Antihypertensives for Treatment-Naïve Patients in South Korea: From Korean NHISS Claim Data

**DOI:** 10.1155/2019/4735876

**Published:** 2019-08-25

**Authors:** Sang Hyuck Kim, Dong Wook Shin, Shinhye Kim, Kyungdo Han, Sang-hyun Park, Yul-Hee Kim, Shin-Ae Jeon, Yong-Chol Kwon

**Affiliations:** ^1^Department of Family Medicine, Bumin Hospital, Seoul, Republic of Korea; ^2^Department of Family Medicine, Samsung Medical Center, Sungkyunkwan University School of Medicine, Seoul, Republic of Korea; ^3^Department of Family Medicine, Gangnam Severance Hospital, Seoul, Republic of Korea; ^4^Department of Biostatistics, College of Medicine, Catholic University, Republic of Korea; ^5^Medical Affairs, Pfizer Essential Health Business Unit, Pfizer Pharmaceuticals Korea Limited, Seoul, Republic of Korea

## Abstract

**Background:**

Several factors influence the choice of antihypertensive drugs. To facilitate the rational use of drugs it is important to assess their prescription patterns over time. This study aims to evaluate doctors' prescribing patterns of antihypertensive drugs for drug-naïve patients in South Korea.

**Methods:**

The claims data of the Korean National Health Insurance Research Database from 1 January 2011 to 31 December 2015 were analyzed. The data virtually cover the entire South Korean population. Antihypertensive drugs were further subdivided into angiotensin receptor blockers (ARBs), angiotensin-converting enzyme inhibitors (ACEis), calcium channel blockers (CCBs), beta-blockers (BBs), and thiazide diuretics. The prescription pattern of antihypertensive drugs and associated factors were assessed according to the patients' characteristics, including associated comorbidities.

**Results:**

A total of 2,919,162 subjects had started taking antihypertension medications during the study period. ARB was the most frequently prescribed drug (51.6%) followed by CCB (45.0%), BB (18.5%), diuretics (17.0%), and ACEi (11.7%). Most patients were prescribed with monotherapy (66.7%) rather than combination therapy (33.3%), and CCB was the most frequently prescribed monotherapy drug (25.7%). For combination therapy, ARB + CCB was the most frequently prescribed combination, and the prescription frequency was found to be increasing. In patients prescribed with combination therapy, most had been prescribed single-pill fixed-dose combination.

**Conclusion:**

We identified the physicians' prescription patterns of antihypertensive drugs for treatment-naïve patients. The findings of this study can lead to a rational, evidence-based, and cost-effective improvement of prescription patterns in newly diagnosed hypertensive patients.

## 1. Introduction

Hypertension is a common chronic condition which is associated with various complications including cardiovascular disease and chronic kidney disease. It remains that hypertension is one of the most important preventable conditions to reduce cardiovascular mortality [1]. Despite this, the high and increasing prevalence of hypertension still contributes to considerable socioeconomic burden globally [2–4].

In order to choose the appropriate antihypertensive drug among various available classes, several factors should be considered, such as age and underlying comorbid conditions [5–7]. Also, the type of antihypertensive drugs can affect prescribing patterns as well as drug compliance [8–11]. Therefore, it is necessary to assess prescription patterns to determine whether current prescription is reasonable, evidence-based, and cost-effective [11]. Although there have been several studies on prescription patterns of antihypertensives in other countries, they are not relevant in the Korean context since prescription patterns are influenced by time and region [9,10,12–14]. Furthermore, previously conducted studies in Korea are either too old or not representative (single hospital-based study) [15, 16].

This study aimed at evaluating prescribing patterns of antihypertensive drugs in patients who were prescribed antihypertensive drugs for the first time.

## 2. Materials and Methods

### 2.1. Data Sources

The claims data of the Korean National Health Insurance Research Database (NHIRD) were analyzed. NHIRD contains qualification data, medical services claim data, and pharmacy claim data. As such, the claims data include patient information such as age, sex, household income, residential regions, as well as diagnosis information (by the International Classification of Diseases, 10th Revision; ICD-10) and specific information of diagnostic tests, procedures, and prescriptions.

In general, the Korean National Health Insurance Service (NHIS) is the sole insurer providing a mandatory universal health insurance which virtually covers the entire Korean population (about 97% of total population) and a medical aid program to those in the lowest income bracket who are covered by government funding. We used the nation-wide claims data which covered the South Korean population over a 5-year period, from 1 January 2011 to 31 December 2015. Detailed insights into the advantages of this data are described elsewhere [17, 18].

### 2.2. Study Population

From the whole Korean population (*N* = ∼50 million), patients who were newly diagnosed with hypertension (ICD-10: I10, I11, I12, I13, or I15) and prescribed with antihypertensive agents were included in this study. We further confirmed that the study subjects did not have any previous record of antihypertensive medication during the prior 12 months.

### 2.3. Variables

#### 2.3.1. Antihypertensive Agents

Antihypertensive agents were classified into 5 major categories, including angiotensin receptor blockers (ARB), angiotensin-converting enzyme inhibitors (ACEi), calcium channel blockers (CCB), beta-blockers (BB), and thiazide diuretics (including indapamide and chlorthalidone), or other antihypertensive drugs.

Initial prescription was divided into single drug and combination. Single drug was defined as the prescription of 1 class of antihypertensive at first prescription. Combination was defined as prescription of 2 or more classes of antihypertensives at first prescription and was further categorized by single-pill fixed-dose drug and free combination (multiple-pill combinations). Commonly used combinations available commercially are specifically listed: ARB + CCB, ARB + thiazide, and ARB + CCB + thiazide.

#### 2.3.2. Comorbidities

Comorbidities were defined by ICD 10 codes: diabetes (ICD 10: E11-14), dyslipidemia (E78), congestive heart failure (I50), coronary heart disease(I20-25), stroke (I60 to I64, I67, and I69), chronic kidney disease (N18), and cancer (CX) during the previous 1 year prior to index date (first prescription of antihypertensive medication). The Charlson comorbidity index was also assessed in the same manner [16].

### 2.4. Statistical Analyses

The baseline characteristics of hypertensive patients who were newly prescribed with antihypertensive medication were described using frequencies and percentages or as means with standard deviation.

Descriptive statistics were calculated for patterns of first prescriptions of antihypertensive medications. The proportion of each antihypertensive class was calculated according to patient characteristics (age, sex, income level, and place of residence), medical characteristics (the Charlson comorbidity index and each comorbidity as defined above), provider characteristics (level of hospital; general hospital vs. hospital vs. clinic), and year of first prescription.

Factors associated with choice of first prescription for hypertension were investigated with a series of multivariate logistic regression analyses including all of the aforementioned characteristics. Outcomes were (1) combination vs. single, (2) choice of each drug class among single drug user (exclusive to each other), (3) choice of each drug class among all patients (not exclusive to each other), and (4) fixed-dose combination vs. free combination for selected combinations. Also, the trends of combination therapy according to the age group, income level, and Charlson's comorbidity index were assessed by calculating the “*p* for trend.”

All analyses were performed using the SAS statistical software (ver. 9.3, SAS Institute., Cary, NC, USA). All tests were two-sided, and statistical significance was defined as a *p* value <0.05.

### 2.5. Ethics Statement

This study was reviewed and approved by the Institutional Review Board of the Samsung Medical Center (IRB No. SMC 2007-07-130). The requirement for informed consent was waived because this study is based on routinely collected administrative or claims data.

## 3. Results

### 3.1. Baseline Characteristics

A total of 2,919,162 subjects had started taking antihypertension medications during the study period. 56.0% of these were male patients. The mean age of the study population was 53.0 (±14.5) years. Over half of the study population resided in metropolitan areas. About 67.9% of subjects had 1 or more comorbidity as per the Charlson comorbidity index. Subjects had various hypertension-related comorbid conditions such as dyslipidemia (27.6%), coronary heart disease (13.8%), diabetes (12.7%), stroke, (4.6%), congestive heart failure (4.1%), and chronic kidney disease (0.93%). The annual incidence rate per year was 1.24% in 2011, 1.20% in 2012, 1.13% in 2013, 1.05% in 2014, and 1.12% in 2015, respectively (Table 1).

### 3.2. Frequency and Trend of the Prescription

Around two-thirds of the study population was prescribed with monotherapy at first prescription, and one-third was prescribed with initial combination therapy (Table 2). CCB was the most frequently prescribed monotherapy drug (25.70%), followed by ARB (24.45%) and BB (11.88%). The proportion of ARB and BB prescriptions increased during the study period, while CCB and ACEI decreased. The ARB + CCB was the most frequently prescribed combination (12.59%) and was found to be increasing, while ARB + thiazide (9.79%) was decreasing during the study period (Table 3). Among the 5 drug classes, ARB was the most frequently prescribed drug (51.61%), followed by CCB (45.03%), BB (18.48%), thiazide diuretics (17.01%), and ACEi (3.11%). Initial drug choice differed by comorbidities, e.g., 64.74% of diabetes patients initiated with ARB, while 54.18% of CHF patients were initiated with beta-blockers. In CKD patients, 59.82% received ARB and 8.34% received ACEi (Table 4).

**Figure 1:**
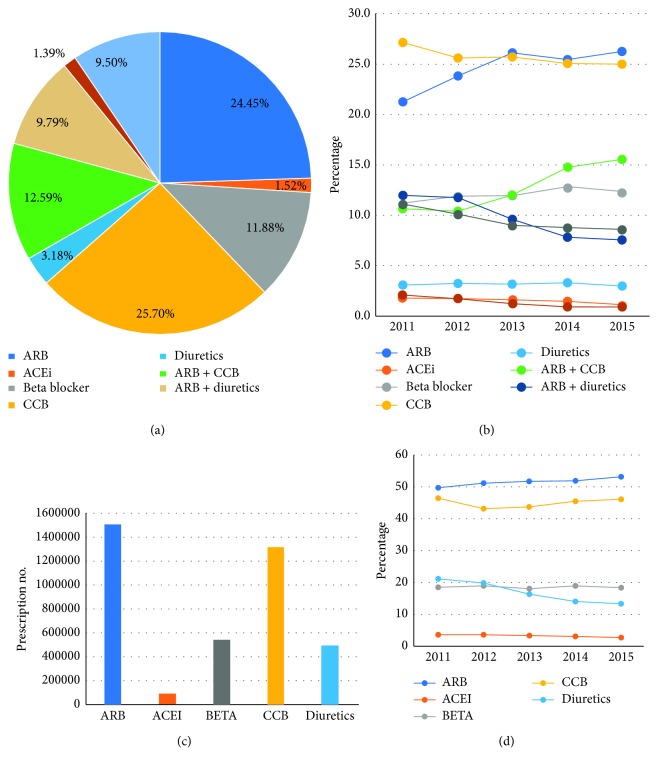
Prescription pattern and trend of the antihypertensive drugs. (a) Prescription pattern including combination therapy, (b) trend including combination therapy, (c) prescription number by drug classes, and (d) trend by drug classes.

A comparison of prescription rates between 2011 and 2015 revealed an increase of 4% in the prescription rate of ARB and an 8% increase in the rate of thiazide prescription (Figure 1).

### 3.3. Trend of Single-Pill Fixed-Dose Combination

Among combination therapy users, most patients were prescribed a single-pill fixed-dose combination (83.73% for ARB + CCB and 96.46% for ARB + thiazide, respectively) in an increasing proportion during the study period (Table 3).

### 3.4. Trend and Factors Associated with Initial Combination Therapy

Female patients were less likely to be prescribed with combination therapy at first prescription (adjusted odds ratio: 0.764, 95% CI 0.760–0.768). Having congestive heart failure, dyslipidemia, CHD, stroke, CKD, and cancer was slightly associated with initiation with combination therapy, while it was opposite with diabetes mellitus. Primary clinics prescribed combination antihypertensive drugs more frequently than secondary hospitals and general hospitals. Initial combination therapy decreased slightly during the study period (35.81 in year 2011 to 32.53% in year 2015). There was no significant trend by the age group or Charlson comorbidity index (Table 2).

## 4. Discussion

In this study, physicians' prescription patterns of antihypertensive drugs for treatment-naïve patients were identified using the National Health Insurance claims data. The strength of this study is its generalizability, as it uses claims data collected from the entire South Korean population and assess data over a 5-year period which allows for the identification of trends in prescription patterns.

In a previous study of prescription patterns in Korea conducted in 2009, CCB was the most frequently prescribed antihypertensive (64.4%), followed by diuretics (44.6%), ARB (33.3%), BB (21.6%), and ACEi (11.7%) [16]. In this study, the corresponding figures were 45.0%, 17.0%, 51.6%, 18.5%, and 3.1%, respectively. This shows that the use of ARB has significantly increased over time, while use of other antihypertensive classes decreased.

Similarly, we also identified that the use of thiazide diuretics and ACEi was decreasing and ARB use was increasing during the study period. Although CCB was the most frequently prescribed drug for monotherapy, overall ARB was the most frequently prescribed class of antihypertensive drugs since the prescription of ARB for combination therapy also increased.

The prescription of CCBs and ARBs increased worldwide over the past several years [11]. Especially, CCBs and ARBs were the most frequently prescribed antihypertensives in the nearby countries of Japan and China [19, 20]. There may be several reasons for this changing prescription trend. The former JNC7 recommended diuretics as the first-line therapy [6, 21], but recent guidelines including regional Korean guidelines announced not only diuretics but also ARBs and CCBs as the first-line treatment, and the corresponding drug utilization appears to have changed [5, 7, 22, 23]. Moreover, although these guidelines included the diuretics as first-line drugs, in East Asian countries including Japan and China, the prescription rate of diuretics for hypertension is lower than that in the United States [19, 20, 24]. Frequent adverse events with the use of diuretics and associated low compliance may account for the low prescription rate [12, 19]. Also, relatively low ACEi prescription presumably reflects the occurrence of frequent side effects in the Asian population such as dry cough [25]. The decreased use of ACEi might account for the increased use of the alternative drug, the ARB. In addition, more favorable adherence and less frequent side effects might increase the use of ARB [12]. Lim et al. had already reported the increase in prescriptions for ARBs and a decrease for diuretics in Korea [15]. However, as the study was based on a single tertiary hospital, the results cannot be generalized to the overall population. Moreover, the study included patients who were already taking antihypertensives, so the proportion of combination therapy was higher than that of the present study (50.9% vs. 33.3%). Although the prescription patterns of antihypertensives differ across countries [11, 19, 20], the proportion of initial combination therapy of the present study seems to be low when compared to nearby countries [26]. Because intensive initial treatment for hypertension is recommended nowadays, an effort to increase the proportion of initial combination therapy may be warranted [5, 27]. Considering patient compliance, a single-pill, fixed-dose combination is a good option [28].

In some groups, other classes rather than CCBs or ARBs were prescribed frequently. In those less than 20 years of age, the use of ACEi and BBs was more frequent. However, it is unclear why ACEi and BBs were used more frequently than in other age groups. Certain underlying conditions such as diabetes, proteinuria, or migraines as well as physician's preference considering adverse drug effects may affect the prescription pattern in children and adolescents [29, 30]. Although several guidelines excluded beta-blocker as a first-line antihypertensive drug [5, 7], the prescription rate of the drug was relatively stable during the study period. It may be due to the presence of comorbid conditions such as ischemic heart disease and arrhythmia, in which the use of beta-blockers is inevitable. However, overall beta-blocker use was found to be relatively low. Unlike the JNC 7 guideline, the JNC 8 guideline did not suggest compelling indications of each antihypertensive drug classes. However, there are several clinical situations which make a specific antihypertensive class preferred to others. For example, JNC 7 and domestic hypertension guidelines in Korea recommend beta-blocker as an initial choice for hypertension in congestive heart failure patients [16]. Therefore, unlike other conditions, beta-blocker may be most frequently prescribed for congestive heart failure patients in the present study, suggesting that clinical guidelines are reflected in prescription of Korean physicians.

On the contrary, we also observed some gaps between the guidelines and clinical practice. According to the JNC 8 guideline, the use of ARB or ACEi was recommended especially in those with chronic kidney diseases [7]. However, the prescription rate of ARB or ACEi in the chronic kidney disease patients was not optimal and was only 68.2%. Even though there can be rational reasons not to use these drugs, a substantial portion of chronic kidney disease patients may lose the chance to prevent or delay disease progression. Doctors often prescribe drugs according to prescription patterns based on their own experience rather than scientific evidences [11]. Further studies are needed to assess and decrease these gaps of prescription pattern.

Overall, the prescription rate of combination therapy was slightly decreasing throughout the study period. In JNC 8 guideline, patients with stage 2 hypertension (BP > 160/100) are recommended to receive combination therapy from the first prescription [7]. In Korea, the national health screening program is offered biennially and more hypertension is diagnosed at an earlier stage due to increasing participation rates [31]. However, considering the relatively low proportion of patients achieving the target blood pressure level (less than 70%) [32], more aggressive treatment for hypertensive patients using combination therapy is warranted.

For initial combination therapy, most patients received single-pill fixed-dose combination drugs and this was an increasing trend. The use of single-pill fixed-dose combination is advantageous in terms of the price of a single-pill combination and the advantage of drug adherence and blood pressure control [6, 7, 11, 33], suggesting a trend toward the favorable prescription pattern.

There are several limitations to be considered in this study. First, there is possibility of false claims of hypertension for reimbursement purpose. For example, beta-blockers are often effective in chronic headache but not reimbursed in Korea for that purpose. So, the physician might have entered the hypertension disease code for making the drug covered by the NHI. Second, we do not have detailed clinical information for each prescription and could not determine the appropriateness of drug choice in individual basis.

## 5. Conclusions

We identified overall prescription patterns of antihypertensives for treatment-naïve patients in South Korea. The findings can lead to a rational, evidence-based, and cost-effective improvement of prescription patterns in newly diagnosed hypertensive patients.

## Figures and Tables

**Table 1:** Baseline characteristics of newly diagnosed hypertension patients (total *n* = 2,919,162)

	*n*	%
Age		
<20	45,222	1.55
20–29	95,489	3.27
30–39	303,523	10.40
40–49	713,524	24.44
50–59	866,370	29.68
60–69	508,662	17.42
70–79	294,771	10.10
≥80	91,601	3.14
Sex		
Male	1,633,768	55.97
Female	1,285,394	44.03
Income		
Medical aid	112,217	3.84
Q1 (low)	766,378	26.25
Q2	696,643	23.86
Q3	663,524	22.73
Q4 (high)	680,400	23.31
Residence		
Metropolitan	1,741,304	59.65
City	833,767	28.56
Rural	344,091	11.79
Charlson comorbidity index		
0	938,134	32.14
1	791,388	27.11
2	497,523	17.04
≥3	692,117	23.71
Provider type		
General hospital	924,592	31.67
Hospital	330,928	11.34
Clinic	1,560,700	53.46
Others (public health centers, etc.)	102,942	3.53
Comorbidity		
Diabetes	371,477	12.73
Dyslipidemia	806,246	27.62
Congestive heart failure	120,749	4.14
Ischemic heart failure	401,547	13.76
Stroke	135,569	4.64
Chronic kidney disease	27,263	0.93
Cancer	44,626	1.53
Year of diagnosis		
2011	627,249	21.49
2012	597,912	20.48
2013	578,479	19.82
2014	538,468	18.45
2015	577,054	19.77

**Table 2:** Prescription pattern by the number of drug classes, *n* (%).

	Single drug class	Combination	Combination (vs. single)
			OR (95% CI)
Overall	1,947,796 (66.72)	971366 (33.28)	
Age (mean ± SD)	53.13 ± 14.48	52.84 ± 14.52	
<20	29,435 (65.09)	15,787 (34.91)	1.214 (1.190–1.239)
20–29	72,099 (75.51)	23,390 (24.49)	0.650 (0.640–0.661)
30–39	202,994 (66.88)	100,529 (33.12)	0.957 (0.948–0.965)
40–49	458,093 (64.2)	255,431 (35.8)	1.094 (1.087–1.101)
50–59	574,198 (66.28)	292,172 (33.72)	1 (ref.)
60–69	348,811 (68.57)	159,851 (31.43)	0.900 (0.894–0.907)
70–79	202,102 (68.56)	92,669 (31.44)	0.913 (0.905–0.922)
≥80	60,064 (65.57)	31,537 (34.43)	1.041 (1.026–1.057)
Sex			
Male	1,048,973 (64.21)	584,795 (35.79)	1 (ref.)
Female	898,823 (69.93)	386,571 (30.07)	0.764 (0.760–0.768)
Income			
Medical aid	75,202 (67.01)	37,015 (32.99)	1.133 (1.118–1.149)
Q1(Low)	499,304 (65.15)	267,074 (34.85)	1.220 (1.212–1.229)
Q2	457,765 (65.71)	238,878 (34.29)	1.184 (1.175–1.193)
Q3	444,104 (66.93)	219,420 (33.07)	1.115 (1.107–1.123)
Q4(High)	471,421 (69.29)	208,979 (30.71)	1 (ref.)
Residence			
Metropolitan	1,172,041 (67.31)	569,263 (32.69)	1 (ref.)
City	549,150 (65.86)	284,617 (34.14)	1.068 (1.062–1.074)
Rural	226,605 (65.86)	117,486 (34.14)	1.077 (1.069–1.086)
Charlson's comorbidity index			
0	616,812 (65.75)	321,322 (34.25)	1.050 (1.041–1.058)
1	531,229 (67.13)	260,159 (32.87)	0.999 (0.992–1.007)
2	338,054 (67.95)	159,469 (32.05)	0.962 (0.955–0.970)
≥3	461,701 (66.71)	230,416 (33.29)	1 (ref.)
Comorbidity			
Diabetes	258,962 (69.71)	112,515 (30.29)	0.816 (0.810–0.823)
Dyslipidemia	528,199 (65.51)	278,047 (34.49)	1.107 (1.101–1.114)
Congestive heart failure	65,091 (53.91)	55,658 (46.09)	1.890 (1.867–1.914)
Ischemic heart failure	257,083 (64.02)	144,464 (35.98)	1.132 (1.123–1.140)
Stroke	87,896 (64.83)	47,673 (35.17)	1.141 (1.128–1.155)
Chronic kidney disease	17,263 (63.32)	10,000 (36.68)	1.193 (1.163–1.224)
Cancer	29,331 (65.73)	15,295 (34.27)	1.177 (1.153–1.201)
Provider type			
General hospital	641,364 (69.37)	283,228 (30.63)	0.767 (0.763–0.772)
Hospital	216,869 (65.53)	114,059 (34.47)	0.964 (0.956–0.971)
Clinic	1019,985 (65.35)	540,715 (34.65)	1 (ref.)
Other	69,578 (67.59)	33,364 (32.41)	0.900 (0.888–0.913)
Year of diagnosis			
2011	402,660 (64.19)	224,589 (35.81)	1 (ref.)
2012	395,541 (66.15)	202,371 (33.85)	0.915 (0.909–0.922)
2013	395,450 (68.36)	183,029 (31.64)	0.824 (0.818–0.831)
2014	364,782 (67.74)	173,686 (32.26)	0.843 (0.837–0.850)
2015	389,363 (67.47)	187,691 (32.53)	0.850 (0.843–0.856)

OR: odds ratio; CI: confidence interval.

**Table 3:** Prescription pattern of antihypertensive monotherapy and combination therapy, *n* (%).

	Monotherapy					Combination therapy						
	ARB	ACEI	BB	CCB	Diuretics	ARB + CCB	Single-pill fixed (ARB + CCB)	ARB + diuretics	Single-pill fixed (ARB + diuretics)	ARB + CCB + diuretics	Separate (ARB + CCB + diuretics)	Others
	713,667 (24.45)	44,406 (1.52)	346,673 (11.88)	750,337 (25.70)	92,713 (3.18)	367,572 (12.59)	307,781 (83.73)	285,869 (9.79)	275,746 (96.46)	40,691 (1.39)	38,083 (93.59)	277,234 (9.50)
Age (mean ± SD)	52.86 ± 12.61	50.34 ± 19.56	49.32 ± 16.68	55.37 ± 13.96	52.69 ± 16.7	51.96 ± 12.56	51.47 ± 12.22	53.99 ± 12.52	53.84 ± 12.41	53.67 ± 13.3	53.8 ± 13.28	52.68 ± 18.43
<20	4,492 (9.93)	4,600 (10.17)	13,417 (29.67)	4,786 (10.58)	2,140 (4.73)	1,227 (2.71)	971 (79.14)	534 (1.18)	510 (95.51)	86 (0.19)	74 (86.05)	13,940 (30.83)
20–29	15,365 (16.09)	1,681 (1.76)	30,155 (31.58)	18,757 (19.64)	6,141 (6.43)	8,426 (8.82)	7,083 (84.06)	4,454 (4.66)	4,279 (96.07)	830 (0.87)	747 (90.00)	9,680 (10.14)
30–39	72,328 (23.83)	4,051 (1.33)	48,706 (16.05)	66,834 (22.02)	11,075 (3.65)	43,945 (14.48)	37,796 (86.01)	25,751 (8.48)	24,977 (96.99)	4,304 (1.42)	3,981 (92.50)	26,529 (8.74)
40–49	194,938 (27.32)	8,606 (1.21)	75,324 (10.56)	160,178 (22.45)	19,047 (2.67)	109,288 (15.32)	94,415 (86.39)	77,024 (10.79)	74,997 (97.37)	11,147 (1.56)	10,387 (93.18)	57,972 (8.12)
50–59	229,509 (26.49)	10,949 (1.26)	87,726 (10.13)	222,731 (25.71)	23,283 (2.69)	114,792 (13.25)	97,355 (84.81)	91,574 (10.57)	88,931 (97.11)	12,316 (1.42)	11,522 (93.55)	73,490 (8.48)
60–69	122,171 (24.02)	7,423 (1.46)	52,154 (10.25)	152,145 (29.91)	14,918 (2.93)	54,608 (10.74)	44,390 (81.29)	51,040 (10.03)	49,072 (96.14)	6,527 (1.28)	6,157 (94.33)	47,676 (9.37)
70–79	60,175 (20.41)	5,136 (1.74)	30,672 (10.41)	94,567 (32.08)	11,552 (3.92)	26,871 (9.12)	20,214 (75.23)	28,025 (9.51)	26,273 (93.75)	3,947 (1.34)	3,749 (94.98)	33,826 (11.48)
≥80	14,689 (16.04)	1,960 (2.14)	8,519 (9.30)	30,339 (33.12)	4,557 (4.97)	8,415 (9.19)	5,557 (66.04)	7,467 (8.15)	6,707 (89.82)	1,534 (1.67)	1,466 (95.57)	14,121 (15.42)
Sex												
Male	420,813 (25.76)	27,129 (1.66)	155,995 (9.55)	417,782 (25.57)	27,254 (1.67)	238,220 (14.58)	200,266 (84.07)	157,969 (9.67)	153,329 (97.06)	25,404 (1.55)	23,701 (93.30)	163,202 (9.99)
Female	292,854 (22.78)	17,277 (1.34)	190,678 (14.83)	332,555 (25.87)	65,459 (5.09)	129,352 (10.06)	107,515 (83.12)	127,900 (9.95)	122,417 (95.71)	15,287 (1.19)	14,382 (94.08)	114,032 (8.87)
Income												
Medical aid	21,158 (18.85)	1,963 (1.75)	19,159 (17.07)	27,747 (24.73)	5,175 (4.61)	10,888 (9.70)	8,507 (78.13)	8,903 (7.93)	8,405 (94.41)	1,477 (1.32)	1,409 (95.40)	15,747 (14.03)
Q1(low)	179,546 (23.43)	10,688 (1.39)	87,246 (11.38)	196,925 (25.70)	24,899 (3.25)	100,997 (13.18)	84,407 (83.57)	78,733 (10.27)	75,970 (96.49)	12,159 (1.59)	11,381 (93.60)	75,185 (9.81)
Q2	167,807 (24.09)	10,021 (1.44)	81,121 (11.64)	176,271 (25.30)	22,545 (3.24)	91,692 (13.16)	77,146 (84.14)	69,714 (10.01)	67,372 (96.64)	10,238 (1.47)	9,573 (93.50)	67,234 (9.65)
Q3	166,039 (25.02)	10,571 (1.59)	75,841 (11.43)	171,715 (25.88)	19,938 (3.00)	83,991 (12.66)	70,344 (83.75)	65,496 (9.87)	63,175 (96.46)	9,085 (1.37)	8,512 (93.69)	60,848 (9.17)
Q4 (high)	179,117 (26.33)	11,163 (1.64)	83,306 (12.24)	177,679 (26.11)	20,156 (2.96)	80,004 (11.76)	67,377 (84.22)	63,023 (9.26)	60,824 (96.51)	7,732 (1.14)	7,208 (93.22)	58,220 (8.56)
Residence												
Metropolitan	441,925 (25.38)	25,693 (1.48)	208,988 (12.00)	443,462 (25.47)	51,973 (2.98)	222,839 (12.80)	189,878 (85.21)	165,196 (9.49)	159,935 (96.82)	22,894 (1.31)	21,283 (92.96)	158,334 (9.09)
City	198,002 (23.75)	12,818 (1.54)	100,108 (12.01)	210,123 (25.20)	28,099 (3.37)	106,064 (12.72)	87,828 (82.81)	84,793 (10.17)	81,631 (96.27)	12,440 (1.49)	11,738 (94.36)	81,320 (9.75)
Rural	73,740 (21.43)	5,895 (1.710)	37,577 (10.92)	96,752 (28.12)	12,641 (3.67)	38,669 (11.24)	30,075 (77.78)	35,880 (10.43)	34,180 (95.26)	5,357 (1.56)	5,062 (94.49)	37,580 (10.92)
Charlson comorbidity index												
0	223,380 (23.81)	7,532 (0.80)	102,265 (10.90)	259,791 (27.69)	23,844 (2.54)	144,663 (15.42)	126,841 (87.68)	101,639 (10.83)	99,032 (97.44)	14,243 (1.52)	13,245 (92.99)	60,777 (6.48)
1	194,677 (24.60)	9,554 (1.21)	93,931 (11.87)	206,751 (26.13)	26,316 (3.33)	101,812 (12.86)	86,629 (85.09)	81,253 (10.27)	78,628 (96.77)	11,177 (1.41)	10,490 (93.85)	65,917 (8.33)
2	125,456 (25.22)	9,257 (1.86)	61,032 (12.27)	124,275 (24.98)	18,034 (3.62)	55,255 (11.11)	45,584 (82.50)	47,316 (9.51)	45,392 (95.93)	6,542 (1.31)	6,118 (93.52)	50,356 (10.12)
≥3	170,154 (24.58)	18,063 (2.61)	89,445 (12.92)	159,520 (23.05)	24,519 (3.54)	65,842 (9.51)	48,727 (74.01)	55,661 (8.04)	52,694 (94.67)	8,729 (1.26)	8,230 (94.28)	100,184 (14.48)
Comorbidity												
Diabetes	146,705 (39.49)	12,888 (3.47)	26,869 (7.23)	65,253 (17.57)	7,247 (1.95)	39,243 (10.56)	31,441 (80.12)	35,983 (9.69)	34,825 (96.78)	4,397 (1.18)	4,140 (94.16)	32,892 (8.85)
Dyslipidemia	240,239 (29.80)	17,020 (2.11)	77,673 (9.63)	176,764 (21.92)	16,503 (2.05)	97,247 (12.06)	79,833 (82.09)	78,273 (9.71)	75,288 (96.19)	10,327 (1.28)	9,564 (92.61)	92,200 (11.44)
Congestive heart failure	14,337 (11.87)	5,768 (4.78)	26,936 (22.31)	14,183 (11.75)	3,867 (3.20)	6,022 (4.99)	3,569 (59.27)	5,859 (4.85)	4,705 (80.30)	964 (0.80)	908 (94.19)	42,813 (35.46)
Ischemic heart failure	58,176 (14.49)	9,904 (2.47)	81,919 (20.40)	99,718 (24.83)	7,366 (1.83)	30,283 (7.54)	19,567 (64.61)	19,949 (4.97)	18,447 (92.47)	4,467 (1.11)	4,238 (94.87)	89,765 (22.35)
Stroke	31,015 (22.88)	2,857 (2.11)	15,783 (11.64)	34,864 (25.72)	3,377 (2.49)	14,745 (10.88)	9,600 (65.11)	8,295 (6.12)	7,727 (93.15)	2,235 (1.65)	2,075 (92.84)	22,398 (16.52)
Chronic kidney disease	8,793 (32.25)	1,102 (4.04)	1,694 (6.21)	5,072 (18.60)	602 (2.21)	3,088 (11.33)	1,769 (57.29)	1,441 (5.29)	1,297 (90.01)	344 (1.26)	324 (94.19)	5,127 (18.81)
Cancer	5,652 (12.67)	651 (1.46)	6,097 (13.66)	16,004 (35.86)	927 (2.08)	3,526 (7.90)	2,498 (70.85)	2,073 (4.65)	1,963 (94.69)	421 (0.94)	400 (95.01)	9,275 (20.78)
Provider type												
General hospital	170,474 (18.44)	27,923 (3.02)	188,819 (20.42)	226,705 (24.52)	27,443 (2.97)	85,950 (9.30)	60,437 (70.32)	25,458 (2.75)	22,832 (89.68)	7,785 (0.84)	6,939 (89.13)	164,035 (17.74)
Hospital	64,955 (19.63)	3,981 (1.20)	38,207 (11.55)	99,156 (29.96)	10,570 (3.19)	50,126 (15.15)	40,075 (79.95)	26,565 (8.03)	25,504 (96.01)	7,847 (2.37)	7,645 (97.43)	29,521 (8.92)
Clinic	457,721 (29.33)	10,968 (0.70)	116,359 (7.46)	382,965 (24.54)	51,972 (3.33)	217,934 (13.96)	197,185 (90.48)	223,409 (14.31)	21,7378 (97.3)	22,652 (1.45)	21,151 (93.37)	76,720 (4.92)
Others	20,517 (19.93)	1,534 (1.49)	3,288 (3.19)	41,511 (40.32)	2,728 (2.65)	13,562 (13.17)	10,084 (74.35)	10,437 (10.14)	10,032 (96.12)	2,407 (2.34)	2,348 (97.55)	6,958 (6.76)
Year												
2011	133,122 (21.22)	11,086 (1.77)	69,095 (11.02)	169,958 (27.10)	19,399 (3.09)	67,370 (10.74)	51,975 (77.15)	74,967 (11.95)	71,990 (96.03)	12,913 (2.06)	12,913 (100)	69,339 (11.05)
2012	142,023 (23.75)	10,293 (1.72)	70,719 (11.83)	153,033 (25.59)	19,473 (3.26)	62,141 (10.39)	48,491 (78.03)	69,941 (11.7)	67,656 (96.73)	10,217 (1.71)	10,217 (100)	60,072 (10.05)
2013	150,777 (26.06)	9,248 (1.60)	68,405 (11.82)	148,626 (25.69)	18,394 (3.18)	68,945 (11.92)	56,772 (82.34)	55,487 (9.59)	53,536 (96.48)	7,025 (1.21)	6,597 (93.91)	51,572 (8.92)
2014	136,533 (25.36)	7,415 (1.38)	68,083 (12.64)	134,755 (25.03)	17,996 (3.34)	79,351 (14.74)	70,132 (88.38)	42,131 (7.82)	40,626 (96.43)	5,123 (0.95)	4,319 (84.31)	47,081 (8.74)
2015	151,212 (26.20)	6,364 (1.10)	70,371 (12.19)	143,965 (24.95)	17,451 (3.02)	89,765 (15.56)	80,411 (89.58)	43,343 (7.51)	41,938 (96.76)	5,413 (0.94)	4,037 (74.58)	49,170 (8.52)

**Table 4:** Prescription pattern by the drug classes, either included in monotherapy or combination therapy, *n* (%).

	ARB	ACEI	BETA	CCB	Diuretics
Overall	150,6561 (51.61)	90,784 (3.11)	539,372 (18.48)	1,314,597 (45.03)	496,634 (17.01)
Age (mean ± SD)	53.01 ± 12.74	53.4 ± 17.74	51.43 ± 16.17	54.32 ± 13.73	54.07 ± 13.81
<20	6,928 (15.32)	5,564 (12.30)	15,015 (33.20)	7,479 (16.54)	3,092 (6.84)
20–29	31,458 (32.94)	2,561 (2.68)	35,064 (36.72)	32,134 (33.65)	13,038 (13.65)
30–39	156,422 (51.54)	7,447 (2.45)	66,968 (22.06)	130,704 (43.06)	48,151 (15.86)
40–49	415,900 (58.29)	17,660 (2.48)	118,228 (16.57)	315,763 (44.25)	124,418 (17.44)
50–59	476,338 (54.98)	24,123 (2.78)	142,677 (16.47)	393,807 (45.45)	148,443 (17.13)
60–69	251,389 (49.42)	17,004 (3.34)	87,818 (17.26)	241,238 (47.43)	86,640 (17.03)
70–79	131,138 (44.49)	11,737 (3.98)	55,469 (18.82)	145,191 (49.26)	54,624 (18.53)
≥80	36,988 (40.38)	4,688 (5.12)	18,133 (19.80)	48,281 (52.71)	18,228 (19.90)
Sex					
Male	904,778 (55.38)	60,192 (3.68)	275,763 (16.88)	775,090 (47.44)	251,286 (15.38)
Female	601,783 (46.82)	30,592 (2.38)	263,609 (20.51)	539,507 (41.97)	245,348 (19.09)
Income					
Medical aid	46,315 (41.27)	3,943 (3.51)	27,160 (24.20)	46,751 (41.66)	19,055 (16.98)
Q1 (low)	398,887 (52.05)	22,550 (2.94)	139,374 (18.19)	353,498 (46.13)	138,079 (18.02)
Q2	363,159 (52.13)	20,640 (2.96)	126,485 (18.16)	315,653 (45.31)	121,217 (17.40)
Q3	346,565 (52.23)	21,268 (3.21)	119,086 (17.95)	299,447 (45.13)	111,549 (16.81)
Q4 (high)	351,635 (51.68)	22,383 (3.29)	127,267 (18.7)	299,248 (43.98)	106,734 (15.69)
Residence					
Metropolitan	909,496 (52.23)	53,311 (3.06)	320,725 (18.42)	778,121 (44.69)	282,095 (16.20)
City	430,908 (51.68)	25,320 (3.04)	156,480 (18.77)	375,042 (44.98)	148,404 (17.80)
Rural	166,157 (48.29)	12,153 (3.53)	62,167 (18.07)	161,434 (46.92)	66,135 (19.22)
Charlson comorbidity index					
0	502,861 (53.60)	12,245 (1.31)	137,490 (14.66)	456,193 (48.63)	163,131 (17.39)
1	411,171 (51.96)	18,536 (2.34)	136,449 (17.24)	355,466 (44.92)	138,614 (17.52)
2	252,680 (50.79)	19,096 (3.84)	97,293 (19.56)	212,683 (42.75)	85,095 (17.10)
≥3	339,849 (49.10)	40,907 (5.91)	168,140 (24.29)	290,255 (41.94)	109,794 (15.86)
Comorbidity					
Diabetes	240,501 (64.74)	21,283 (5.73)	53,265 (14.34)	127,538 (34.33)	55,124 (14.84)
Dyslipidemia	465,523 (57.74)	44,577 (5.53)	155,768 (19.32)	331,832 (41.16)	124,074 (15.39)
Congestive heart failure	45,540 (37.71)	22,783 (18.87)	65,416 (54.18)	37,246 (30.85)	16,936 (14.03)
Ischemic heart failure	148,348 (36.94)	40,612 (10.11)	161,776 (40.29)	178,876 (44.55)	44,121 (10.99)
Stroke	67,621 (49.88)	6,568 (4.84)	34,636 (25.55)	66,925 (49.37)	19,360 (14.28)
Chronic kidney disease	16,310 (59.82)	2,274 (8.34)	5,974 (21.91)	12,220 (44.82)	3,257 (11.95)
Cancer	13,353 (29.92)	1,531 (3.43)	11,312 (25.35)	24,520 (54.95)	4,385 (9.83)
Provider type					
General hospital	350,910 (37.95)	67,357 (7.29)	316,088 (34.19)	406,074 (43.92)	81,064 (8.77)
Hospital	161,403 (48.77)	5,958 (1.80)	57,090 (17.25)	176,623 (53.37)	55,586 (16.80)
Clinic	946,054 (60.62)	14,843 (0.95)	160,542 (10.29)	668,985 (42.86)	339,547 (21.76)
Others	48,194 (46.82)	2,626 (2.55)	5,652 (5.49)	62,915 (61.12)	20,437 (19.85)
Year					
2011	312,074 (49.75)	21,853 (3.48)	115,374 (18.39)	291,626 (46.49)	132,317 (21.09)
2012	306,353 (51.24)	20,206 (3.38)	112,748 (18.86)	257,977 (43.15)	117,653 (19.68)
2013	300,076 (51.87)	18,703 (3.23)	104,476 (18.06)	252,941 (43.73)	94,175 (16.28)
2014	280,036 (52.01)	15,514 (2.88)	101,408 (18.83)	245,769 (45.64)	76,045 (14.12)
2015	308,022 (53.38)	14,508 (2.51)	105,366 (18.26)	266,284 (46.15)	76,444 (13.25)

## Data Availability

The data are available at the online public repository of the National Health Insurance Sharing Service (https://nhiss.nhis.or.kr/bd/ay/bdaya001iv.do). As the data are owned by the National Health Insurance, institutional approval must precede before providing the dataset.
